# Mechanisms and physicochemical characteristics of modified starches in shrimp (*Penaeus vannamei*) myofibrillar protein gels

**DOI:** 10.1016/j.fochx.2026.103496

**Published:** 2026-01-05

**Authors:** Jie Mi, Miao Jin, Gaoshang Li, Dalun Xu, Ru Jia, Huamao Wei, Xiaojun Zhang, Guangming Mei, Wenge Yang

**Affiliations:** a Zhejiang Key Laboratory of Intelligent Food Logistic and Processing, Zhejiang-Malaysia Joint Research Laboratory for Agricultural Product Processing and Nutrition, College of Food Science and Engineering, Ningbo University, Ningbo 315211, China; b Ningbo Joysun Product Testing Service Co., Ltd., Ningbo 315048, China; c Zhejiang Marine Fisheries Research Institute, Zhoushan 316021, China

**Keywords:** Acetylated distarch adipate, Hydroxypropyl distarch phosphate, Myofibrillar protein, *Penaeus vannamei*, Gelation, Molecular docking

## Abstract

In order to explore the theoretical basis for the development of shrimp surimi products, the effects of acetylated distarch adipate (ADA) and hydroxypropyl distarch phosphate (HPDSP) on the shrimp (*Penaeus Vannamei*) myofibrillar protein gel (MPG) properties were studied using dynamic rheological, structural characteristics and molecular docking. The results showed that the addition of ADA or HPDSP performed well in dynamic rheological and texture profile analysis. Meanwhile, 1.0 % ADA and 1.0 % HPDSP had a homogeneous microstructure, hydrophobic interactions would affect the textural properties of MPG and Leu 62, Ser 64, Arg 51 may be the major binding sites for ADA, HPDSP and shrimp myosin. These findings provide a theoretical foundation for utilizing modified starches to improve the quality of high value shrimp surimi products.

## Highlights

• The presence of novel starches could improve the quality of gels.
• The shape of novel starches at 1.0 % addition was regular and uniform.
• The β-sheet and random coil structure was important in gels.
• The pore size in gels was correlated with adhesiveness and cohesiveness.
• The Leu 62, Ser 64, Arg 51 may be the major binding sites for novel starches and myosin.

## 1 Introduction

Myofibrillar protein (MP) plays an important role in surimi products and it can determine the quality of gel products. It has been considered that the gel-forming ability and viscoelasticity of surimi products are attributed to the protein network interactions. Generally, the specific coagulability and protein structure of shrimp surimi might generate a weaker gel-forming ability (Tammatinna, Benjakul, Visessanguan, & Tanaka, 2007), compared with other fish surimi products. And the weak gel strength can lead to products with undesirable texture, high cooking loss, and poor formability, which ultimately results in increased product rejection rates, reduced consumer acceptance, and significant economic waste for the industry. However, consumers prefer to buy shrimp surimi products for its unique flavor. *Penaeus Vannamei* (*P. vannamei*) is one of the important economic aquatic products in China with increasing production year by year, and has reached 2.24 million tons in 2023 according to the Ministry of Agriculture Fisheries and Fisheries Administration. Recently, more and more *P. vannamei* have been used directly in the production of smooth and delicious shrimp surimi products to satisfy the growing demand of consumers (Song et al., 2022; Yang et al., 2020; Zhang et al., 2022). Therefore, the improvement of the gel network of shrimp MP is a central issue to enhance the quality of shrimp surimi products.

There have been a great number of studies focused on the application of starches to surimi products (Liu et al., 2022; Mi et al., 2021) and reported that starch could reduce the production costs by replacing part of the protein without reducing the quality of the surimi (Hunt, Getty, & Park, 2009). In addition, starch also has significant salt-reducing potential in surimi products, which is a key trend in the development of healthy foods. Owing to its excellent water absorption and swelling properties, starch can improve water-holding capacity and gel strength without relying on salt, thus offering a promising strategy for partially replacing salt without affecting the gel quality of surimi products. Modified starches have better gel quality improvement ability compared with native starch. In terms of improving the gel strength of cod surimi, the modified starch such as cross-linked hydroxypropylated cassava starch (CHCS) or cross-linked acetylated cassava starch (CACS) was superior to native cassava starch (Kong et al., 2016). On the other hand, acetylated distarch phosphate (ADSP) has strong cross-linking properties and suitable water absorption, the addition of 10 % ADSP maximized the hardness and chewiness of soybean protein gel (Yu, Ren, Zhao, Cui, & Liu, 2020). In surimi products, modified starch mainly served as the additive. It improved the quality of surimi products by enhancing texture and processing performance; it could also improve the 3D printing adaptability of surimi products to enhance the nutritional and functional properties of the products (Liu et al., 2025, Liu et al., 2025; Li et al., 2025; Wang et al., 2025).

The effects and mechanism of modified starches on MPG suggested that the limited swelling property contained in modified starches could absorb water and compress the MP three-dimensional networks, thus improving the stability of water, reducing the emergence of water channels in the gel network, and promoting the formation of compactness and integrity of the gel network (Fan et al., 2019; Zhao et al., 2022, Zhao et al., 2022; Zhuang, Wang, Jiang, Chen, & Zhou, 2020). However, the interaction between different kinds of modified starches and different sources of MP is diverse and unique. Acetylated distarch adipate (ADA) is modified through acetylation and cross-linking with adipic acid, which is an excellent stabilizer and thickener. Hydroxypropyl distarch phosphate (HPDSP) is modified via hydroxypropylation and cross-linking with phosphate, which improves the paste with strong freeze-thaw stability and transparency. The unique chemical modification of modified starches has led to their wide application in meat products. Several transitions during gelation could be detected via dynamic rheological which contributed to explaining the changes in gel properties caused by proteolysis (Rawdkuen, Benjakul, Visessanguan, & Lanier, 2008). Raman spectroscopy has been applied in the determination of protein structure to elaborate the protein-protein interaction, protein aggregation and protein conformation (Rygula et al., 2013). In addition, the microstructure of protein gels, including the distribution of starch granules and the pore structure of protein networks, could be observed using a light microscope.

In our previous studies, the effect of ADA and HPDSP on physicochemical properties (chemical bonds, water state, gel strength and WHC) of shrimp MPG has been reported (Mi et al., 2022a; Mi et al., 2022b). However, the research on dynamic rheological and structural characteristics of shrimp MPG is scarce. Therefore, the objective of this study is to explore the mechanism of ADA and HPDSP affecting *P. vannamei* MPG formation using texture profile analysis (TPA), dynamic rheological determination, microstructure observation and protein structure measurement. The results will provide the theoretical basis for the development of shrimp surimi products.

## 2 Materials and methods

### 2.1 Materials

Live shrimps (*P. vannamei*), which had a weight of 12.87 ± 1.46 g and a length of 12.80 ± 0.48 cm, were purchased from Lulin market (Ningbo, Zhejiang, China) and transported to the laboratory within 2 h in ice. After deveining the shrimps, each 100 g of shrimp meat was vacuumed in a polytetrafluoroethylene bag and stored in a freezer at −40 °C until use. ADA and HPDSP were supplied by Starpro Company (Hangzhou, China).

### 2.2 Extraction of shrimp myofibrillar protein

The extraction of MP from shrimp meat was performed according to the method of Li et al. (2020). Frozen shrimp meat was thawed at 4 °C for 10 h. A 5 g of thawed shrimp meat was homogenized with four times (*w*/*v*) of buffer A (50 mM PBS, containing 2 mM MgCl_2_, 1 mM EDTA and 0.1 M NaCl, pH 7.0), then centrifuged (4 °C，8000 rpm, 15 min) and rewashed twice with the same buffer. After that, the precipitate was mixed with buffer B (50 mM PBS containing 0.1 M NaCl) and centrifuged (4 °C, 8000 rpm, 15 min). Finally, the precipitate was myofibrillar protein (MP). Adjusting the protein concentration to 70 mg/mL with buffer B for subsequent experiments using Lowry's method with bovine serum albumin as the standard.

### 2.3 Addition of modified starch in MP

To ensure the ratio of protein to water remained constant in all samples, a predetermined mass of water (calculated to achieve the target final MP concentration) was first used to fully disperse the modified starch. The starch was then gradually added to the MP solution and thoroughly mixed to ensure uniform distribution. ADA and HPDSP were added to the MP solution at 0.5 %, 1.0 %, 1.5 %, 2.0 %, and 2.5 % (*w*/w, starch/protein), respectively. The group without ADA and HPDSP was the control group. The MP samples were all used for dynamic rheological tests and preparation for myofibrillar protein gel (MPG).

Each group of MP was heated at 90 °C for 30 min in a water bath, then cooled in an ice bath for 30 min to obtain the myofibrillar protein gel (MPG), which is then stored at 4 °C for subsequent analysis. The MPG samples were prepared for texture test, light microscopy observation and Raman spectroscopy test.

### 2.4 Dynamic rheological properties of MP

Dynamic rheological tests were mainly used to simulate the processing conditions. The temperature sweep simulated the entire heating process during gel preparation, while the frequency sweep and strain tests evaluated the mechanical stability of the gel under different deformation rates and stresses. Dynamic rheological tests were performed on the MP samples according to the method of Gani and Benjakul (2018) using a Dynamic Shear Rheometer (DHR-2 Discovery hybrid rheometer, TA Instrument, USA) with a slight modification. A 40 mm parallel steel plate was used for temperature and frequency sweep measurements, respectively. Silicone oil was applied to prevent the evaporation of water. The gap between the parallel steel plate and MP samples was adjusted to 1 mm. The MP samples were gradually heated from 4 to 90 °C (2 °C/min), accompanied by a strain of 1 % and a frequency of 1 Hz. Then cool down from 90 °C to 4 °C at a rate of 5 °C/min, maintain at 4 °C for 10 min, and set the frequency scan to 0.1 Hz to 100 Hz while maintaining 1 % strain. Storage modulus (G′), loss modulus (G′′) and loss tangent (tan δ) were recorded, respectively.

### 2.5 Texture profile analysis of MPG

Texture profile analysis (TPA) was performed using a texture analyzer (TA. XT. Plus, Stable Micro Systems, Surrey, UK) as described by An et al. (2021). The MPG samples were compressed twice with a cylinder probe (P 36R, 36 mm diameter). The conditions for TPA were as follows: 5.0 mm/s of pre-test speed, 1.0 mm/s of test speed; 5.0 mm/s of post-test speed; 5.0 g of trigger force; and 50 % compression. Cylindrical samples (2 cm diameter × 2.5 cm height) were prepared.

### 2.6 Light microscopy observation of MPG

According to the method of Jia et al. (2020), the frozen MPG were cut into 5 mm cubes and immersed in a fixative solution (formaldehyde, diluted with 9 times ethanol) for more than 24 h at −20 °C. The samples were subsequently dehydrated with gradient alcohol as well as xylene and embedded in paraffin. A 4 μm thick sample was cut and stained with periodic acid-schiff (PAS) or hematoxylin-eosin (HE). The slides were observed with a light microscope (Model Eclipse Ts2R-FL, Nikon Corporation, Japan). Nano measurer software (Nano measurer 1.2.5, Fudan University, Shanghai, China) was used to measure the particle size of modified starch and pore size in MPG.

### 2.7 Raman spectroscopy of MPG

Raman determination was described as the method of Lina, Yanshun, Qixing, Wenshui, and Chunjiang (2014) using Renishaw in Via Reflex (Renishaw, UK). Approximately 1 mm slice of MPG sample was placed under the lens and then regulating the excitation light source at 785 nm to sweep the spectra range of 500 to 3000 cm^−1^. The accumulation, grating, exposure time and objective were 19,1200 I/mm, 10 s and 50 × magnification, respectively. The smooth corrected, baseline corrected and normalized Roman Spectra was analyzed by Peakfit 4.12 (SeaSolve Software Inc., California, the USA). The Amide *I* bond was used to fit the secondary structure proportions of MPG samples (Zhang, Yang, Zhou, Zhang, & Wang, 2017).

### 2.8 Molecular docking of myosin and HPDSP, ADA

#### 2.8.1 Model acquisition

The structure of ADA (PubChem SID 446407076), HPDSP (PubChem SID: 441151748) and the amino acid sequence of *P. vannamei* myosin (GenBank: ROT75475.1) were obtained from National Center for Biotechnology Information.

#### 2.8.2 Homology modeling of myosin

Homology modeling of myosin was conducted by Alphafold, the Ramachandran plot and ERRAT were used to evaluate the modeling results by SAVES 6.0 (Jiang et al., 2024).

#### 2.8.3 Molecular docking

The docking studies were conducted by Autodock Vina. The model obtained in 2.8.1 as the ligand and the model obtained in 2.8.2 as the receptor during the process of molecular docking. The ligand and receptor were subjected to water removal and hydrogenation. Then, the grid box was used to ensure the position of receptor (in box) and ligand (out box) before the running of docking program. The docking results with the highest scores were further analyzed. The 3D visualization was carried out using PyMol.

### 2.9 Statistical analysis

All experiments were replicated thrice and the results were expressed as means ± SD. Analysis of variance was performed using SPSS 25.0 statistical software (International Business Machines Corporation, Armonk, New York, USA). Duncan's method was applied and *p* < 0.05 was considered a statistically significant difference.

## 3 Results and discussion

### 3.1 Dynamic rheological properties

**Fig. 1:**
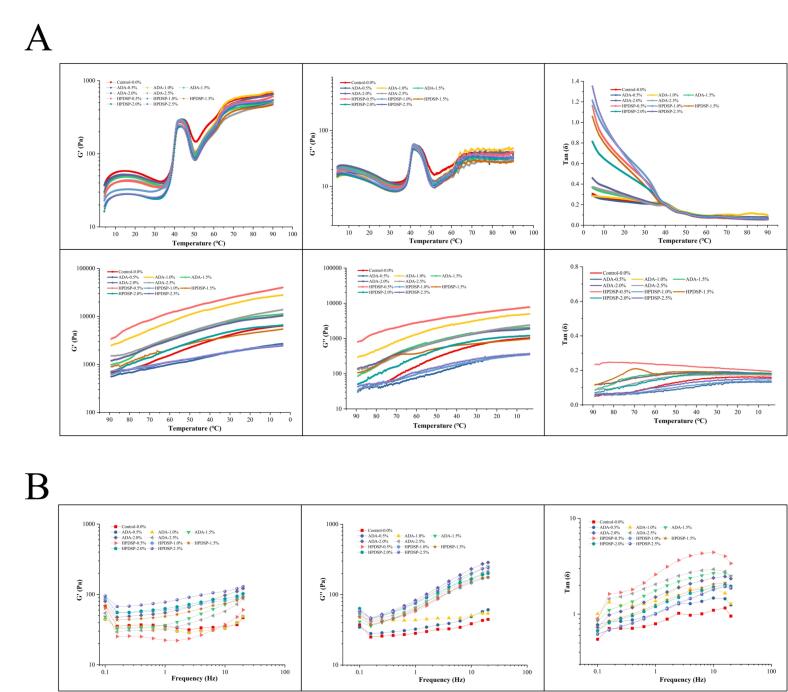
Effects of ADA and HPDSP addition on the dynamic rheological properties of MP (A) the G' value, G" value, tan δ value in temperature sweep; (B) the G' value, G" value, tan δ value in frequency sweep.

Thermal promotes protein aggregation with the formation of dense three-dimensional gel networks (Jia, Lin, Zhang, Zheng, & Liu, 2022). Therefore, the mechanism of gel formation could be revealed by using dynamic rheological analysis to detect the changes in proteins during the heating process. In general, the storage modulus (G′) is the energy stored in the gel during shear and reflects the elastic properties of the gel, while the loss modulus (G″) refers to the viscous property of the gel (Zhou et al., 2020). The G″/G′ was expressed as tan δ. The result in Fig. 1A shows that the G′ of the control group and ADA group increased from 35 °C to 43 °C, then declined sharply to the trough at 51 °C and increased steadily during the subsequent heating. Whereas the G′ of HPDSP group began to increase at 30 °C lower than that of control group and ADA group, indicating that HPDSP has strong water swelling even at low temperatures. The G′ value peaked at 43 °C for all groups, suggesting that significant structural changes and myosin interactions were occurring. A similar phenomenon was observed during the preparation of surimi by Luo et al. (2020). Subsequently, the shrimp MP underwent gel degradation at 43–50 °C due to high endogenous serine enzyme activity. However, the inhibition of endogenous serine enzyme activity by ADA and HPDSP was not significant, and both had high G' values at high temperatures (50–90 °C). As shown in Fig. 1A, the G′ value of all groups increased rapidly during cooling process. The highest G′ value was observed in the 0.5 % HPDSP group, followed by the 1.0 % ADA group, indicating that both modified starch could enhance the gel formation ability of MPG during the cooling process. Compared with the control group, the addition of ADA or HPDSP did not significantly increase the storage modulus (G') during the heating stage (50–90 °C). This indicated that modified starch did not directly accelerate or enhance the heat-induced gelation of myosin itself. Instead, their main functions during the heating process seemed to be hydration and swelling, and integrating into the protein matrix as it unfolded and aggregated. The significant difference in G' observed during the cooling stage (Fig. 1B) emphasized their key role in determining the final gel strength.

The tan δ value was used to evaluate the properties of the gel. The gel behaves as viscous when the tan δ value is higher than 1, otherwise, the gel shows elasticity (Chen, Zhang, & Yang, 2021). As shown in Fig. 1A, the tan δ values of the HPDSP groups were higher than those of ADA and control groups. The increase in viscosity of HPDSP group maybe due to the increasing gelatinization rate (Mi et al., 2022b). Whereas the hydrophilic groups of ADA allowed it to bind more water molecules for better swelling ability.

As shown in Fig. 1A, the higher G″ values of the HPDSP groups were observed at low temperatures (20–50 °C), and the 0.5 % HPDSP group showed higher G″ values during the cooling process. Moreover, all groups exhibited elastic properties during cooling process (Fig. 1A), suggesting that cooling could contribute to the formation of hydrophobic interactions in the gels.

Poowakanjana, Park, Moon, and Yoon (2015) reported that the G′ value of surimi paste showed the typical viscoelastic properties in the low-frequency sweep range from 0.1 to 10 Hz, and became stable at high temperature, which was consistent with the result of Fig. 1B. Besides, The G' values of 0.5 % ADA group, 1.0 % ADA group and control group were more stable than the other groups. The G' value of HPDSP was related to frequency and has a significant impact on the performance of MPG. It could be concluded that ADA could resist the frequency and improve the stability of MPG, which was meaningful for storage, transportation and distribution. In conclusion, the presence of ADA or HPDSP could result in significant changes in the properties of MPG, and both of them could improve the quality of MPG with different conditions. The distinct dynamic rheological properties of ADA and HPDSP groups suggested their different roles in network formation. ADA enhanced the G′ during the later heating and cooling stages, while HPDSP showed a significant increase in G″ at lower temperatures. This indicated that HPDSP, with its hydroxypropyl and phosphate groups, may engage in earlier and stronger interactions with water and partially unfolded myosin, altering the initial aggregation pathway.

### 3.2 Texture profile analysis

As an important characteristic of gel products, texture determines the popularity and economic value of products, and TPA is a significant indicator to characterize the sensory properties of cooked surimi products. The hardness, adhesiveness, springiness, cohesiveness, chewiness, and resilience of MPG are shown in Table 1. Compared with the control group, the hardness of MPG increased significantly with the addition of ADA, and the highest hardness (349.74 ± 25.63 g) was observed at 0.5 % addition. The adhesiveness scores improved in ADA and HPDSP groups compared with control group. While the 0.5 % HPDSP group performed the higher adhesiveness score (−28.03 ± 3.74 g*sec), which was consistent with the results of the rheological result (Fig. 1A). When at higher addition (1.5 %), competition for water becomes intense and the limited swelling space leads to incomplete granule swelling and potential starch-starch interactions, which can disrupt the continuous protein network and thereby reduce hardness.

**Table 1:** Effect of ADA and HPDSP addition on the texture profile analysis of MPG.

Addition (%)		Hardness (g)	Adhesiveness (g*sec)	Springiness	Cohesiveness	Chewiness (g)	Resilience
Control	0.0	195.50 ± 14.61^ef^	−11.43 ± 4.27^d^	0.87 ± 0.02^bc^	0.72 ± 0.01^c^	123.74 ± 7.69^g^	0.41 ± 0.01^e^
ADA	0.5	349.74 ± 25.63^a^	−16.48 ± 2.15^bcd^	0.89 ± 0.05^ab^	0.76 ± 0.02^ab^	236.80 ± 20.10^a^	0.45 ± 0.01^d^
	1.0	299.59 ± 28.89^b^	−22.54 ± 2.67^ab^	0.93 ± 0.01^a^	0.78 ± 0.02^a^	217.21 ± 18.57^abc^	0.47 ± 0.00^abc^
	1.5	319.50 ± 22.45^ab^	−20.43 ± 3.26^bcd^	0.93 ± 0.03^ab^	0.77 ± 0.01^ab^	228.39 ± 22.61^ab^	0.47 ± 0.01^ab^
	2.0	286.65 ± 17.92^bc^	−16.23 ± 1.41^bcd^	0.92 ± 0.02^ab^	0.78 ± 0.00^a^	205.02 ± 13.27^bcd^	0.48 ± 0.00^ab^
	2.5	256.53 ± 11.53^cd^	−20.72 ± 2.05^abc^	0.91 ± 0.04^ab^	0.78 ± 0.02^a^	183.15 ± 11.23^de^	0.48 ± 0.01^a^
HPDSP	0.5	187.04 ± 26.60^f^	−28.03 ± 3.74^a^	0.88 ± 0.03^ab^	0.72 ± 0.01^c^	119.5 ± 20.93^g^	0.42 ± 0.00^e^
	1.0	291.44 ± 10.11^bc^	−15.23 ± 3.27^bcd^	0.92 ± 0.00^ab^	0.74 ± 0.00^bc^	200.93 ± 6.02^bcd^	0.45 ± 0.00^cd^
	1.5	241.71 ± 42.90^d^	−15.21 ± 2.90^bcd^	0.90 ± 0.01^ab^	0.72 ± 0.02^c^	157.02 ± 22.17^ef^	0.44 ± 0.01^d^
	2.0	293.00 ± 17.73^bc^	−15.90 ± 2.66^bcd^	0.90 ± 0.00^ab^	0.74 ± 0.01^bc^	196.19 ± 7.29^cd^	0.45 ± 0.00^bcd^
	2.5	233.06 ± 11.28^de^	−13.33 ± 2.05^cd^	0.90 ± 0.00^ab^	0.72 ± 0.01^c^	152.78 ± 11.34^f^	0.45 ± 0.00^d^

Values are presented as mean ± SD. Different letters in the same column mean significant difference (*p* < 0.05).

The higher springiness values in the 1.0 % ADA, 1.5 % ADA and 1.0 % HPDSP groups indicated that ADA and HPDSP positively affected the quality of MPG. Kim and Lee (1987) found that the swelling of starch granules would exert pressure on gel matrix and resulting in a network denser and firmer. Wang, Tu, Shi, and Yang (2022) indicated that adding more than 4 % (*w*/w) okara to surimi would lead to a decrease in the elasticity of the surimi gel. This was because the increased binding of okara to water molecules displaced the protein-water interactions.

Cohesiveness is defined as the area of the first compression divided by the same area before the peak force of the second compression during the test period. The cohesiveness values of the ADA groups were greater than those of the HPDSP groups at same addition, which was closely related to the granule swelling of the starch. The larger the pore size, the more free water was present in the protein structure, resulting in ADA or HPDSP particles not being well embedded in the protein network structure, leading to an increase and decrease in adhesiveness, respectively. So the relationship between modified starch, protein and free water should be considered.

The acetyl groups in ADA were predominantly hydrophobic in character. When ADA swelled, these hydrophobic moieties were exposed and likely to engage in hydrophobic interactions with MP. These interactions could reinforce the gel structure internally and enhance the ability of the gel to withstand deformation, which contributed to a more integrated, continuous structure, leading to higher cohesiveness. In contrast, HPDSP contained hydroxypropyl and phosphate groups, which were highly hydrophilic. While HPDSP also swelled and filled the protein matrix, its primary interaction with the system was manifested in strong competition for water molecules and the formation of extensive hydrogen bonds with water. These resulted in the distinctions of cohesiveness due to the different influence mechanisms of structures on protein networks.

**Fig. 2:**
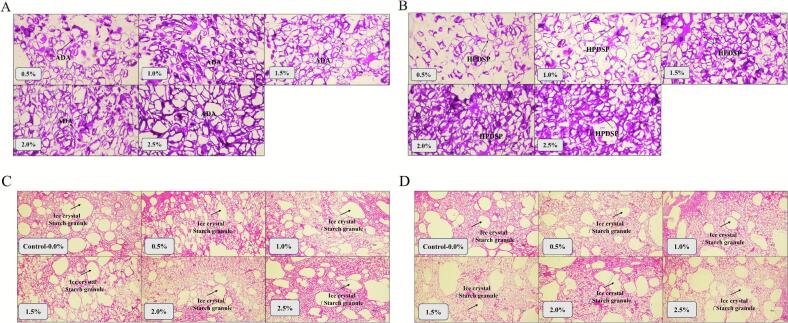
Effects of ADA and HPDSP addition on the micrograph of MPG. (A) the shape of ADA in MPG (20× magnification); (B) the shape of HPDSP in MPG (20× magnification); (C) effects of ADA on the shape of ice crystal in MPG (4× magnification); (D) effects of HPDSP on the shape of ice crystal in MPG (4× magnification).

**Fig. 3:**
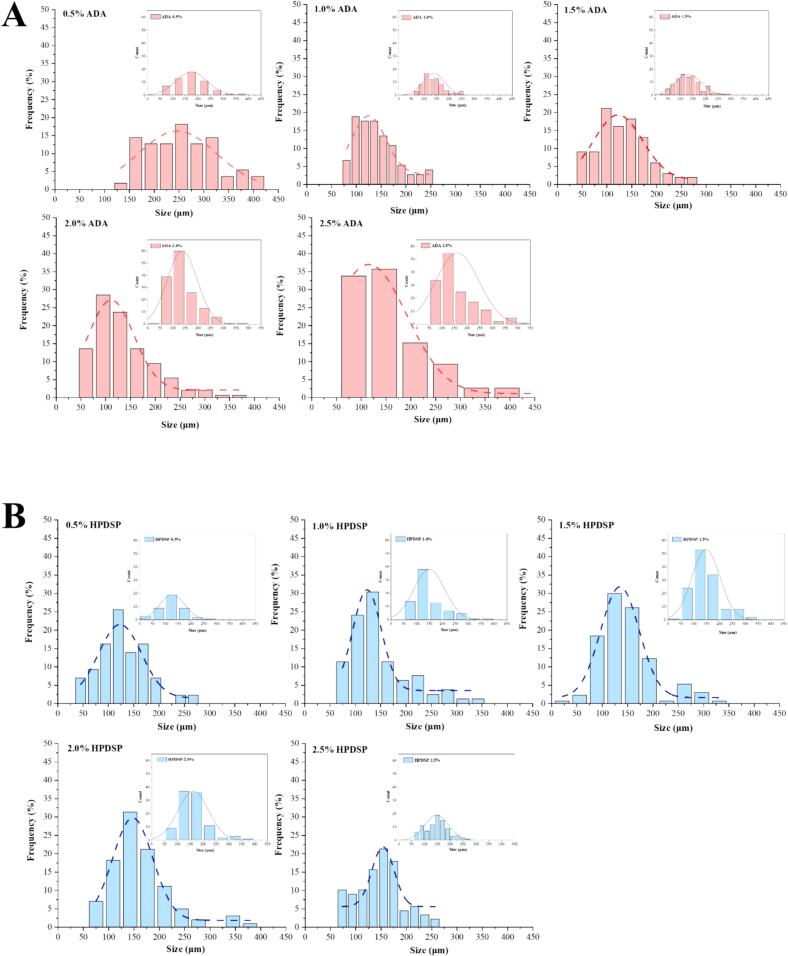
The normal distribution curve of modified starch particles. (A) the normal distribution curve of ADA particle added in the MPG. (B) the normal distribution curve of HPDSP particle added in the MPG.

The gelatinization and swelling ability of starch granules might improve the hydration of starch granules, resulting in better compatibility between the protein and starch granules, and hence improve cohesiveness and resilience of surimi. Wijayanti, Singh, Benjakul, and Sookchoo (2021) suggested that cohesiveness is an important force within chemical bonds in the product structure, and might form strong chemical bonds in protein chains. Therefore, samples with higher cohesiveness may have better TPA, which indicated that their internal network structure was tightly connected, uniform, and not easily damaged. In conclusion, the 1.0 % ADA and 1.0 % HPDSP groups showed better texture properties than the control group. ADA favored hardness and cohesiveness, whereas HPDSP enhanced adhesiveness. This functional divergence could be linked to their microstructural impact (Fig. 2, Fig. 3). ADA formed regular, swelling particles that reinforced the network, while HPDSP's uniform distribution and limited swelling likely promoted a finer, more continuous protein-starch interface.

### 3.3 Light micrograph observation

The modified starch granules were visible in purple color at 20× magnification in Fig. 2A, B and formed a spherical morphology during the heating process. The normal distribution curve of ADA and HPDSP particles in the MPG was shown in Fig. 3, and the mean size of modified starch (ADA and HPDSP) in MPG was shown in Table S1.

In the 1.0 % ADA group, the particle size was mainly distributed between 100 and 150 μm. And in the 1.0 % HPDSP group, the particle size was mainly distributed in 125 μm which was more concentrated and homogeneous than that in ADA groups. The mean size of ADA-0.5 % was the biggest (184.35 ± 63.78 μm) among all groups, which maybe attributed to its ability to rapidly obtain water molecules and swell sufficiently. When ADA swells sufficiently, it exhibited strong hardness (Table 1). However, with the increasing addition of ADA (1.0 % ∼ 2.0 %), the particle size seems to reach equilibrium. And this balance was broken at the addition of 2.5 %, the strong competition phenomenon began to occur among starch particles. The change in particle size of HPDSP was similar to this situation. One minor difference was that the particle size of HPDSP was smaller (127.36 ± 48.37 μm) and the water-absorbing capacity was not as strong as that of ADA when the addition amount of HPDSP was 0.5 %, which might be due to the different basic properties of starch.

With the increasing addition of ADA and HPDSP, the starch particles exhibited oval or irregular shapes due to reduced swelling degree. The starch granules could act as fillers to fill the protein matrix and put pressure on the gel structure when they absorbed enough water and swelled sufficiently. And the competition between starch-protein, and starch-starch for water would inhibit the swell of starches (Zhao et al., 2022, Zhao et al., 2022). Jiang et al. (2021) reported that small spherical granules of starch contributed to filling into the surimi matrix. The results showed that the nearly spherical morphology of starch granules in 1.0 % ADA and 1.0 % HPDSP groups had an excellent ability to absorb water and fill into the gel matrix completely to support the network structure, the similar phenomenon was observed by Jia et al. (2018).

The protein was visible in red color at 4× magnification, the pores of water molecules and the muscle fiber were marked with white and red, respectively (Fig. 2C, D). The pores in the 1.0 %ADA group and 1.0 % HPDSP group was relatively more regular and homogeneous, but as the amount of modified starches increased, large pores formed and extruded the muscle fibers.

Large amounts of modified starch absorbed excess water molecules and promoted the pore's growth and enlargement (Gong et al., 2020). In addition, the enlarged pores would also exert greater pressure on the modified starch, which resulted in a large extent of damage to the modified starch granules. The mean size of pores in MPG is shown in Table S1, the granules from the ADA-0.5 % group had the smallest pore size in the MPG (157.15 ± 42.81 μm). With the increase of ADA (1.0 %–2.0 %) addition, the pore size became larger, and the particle size of ADA became smaller. The effect of HPDSP addition growth seemed to be more stable but the hardness was weaker (Table 1). Maybe the limited swelling ability of HPDSP could promote the MPG structure stronger. It is possible that the limited swelling ability of HPDSP could promote the MPG structure stronger. Additionally, the ADA group could reduce more pores in MPG compared with the control group.

The results in Fig. 2C and Fig. 2D showed that the pores in the control group were more uniform in shape, and the pores might the be starch granules or ice crystal, while the springiness was weaker than the other groups (Table 1). The structure of the protein gels has elasticity and firmness to tolerate the pressure caused by the expansion of modified starch. The proper addition of modified starch could fill into the protein network fully to support the protein structure (Hunt et al., 2009). Therefore, 1.0 % ADA or 1.0 % HPDSP could effectively improve the characteristics of MPG.

### 3.4 Raman spectroscopic analysis

**Fig. 4:**
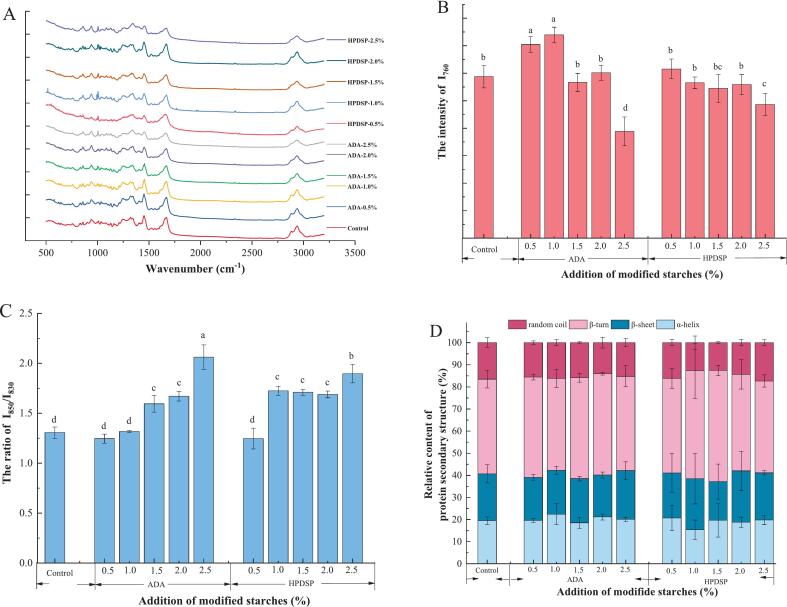
Effects of ADA and HPDSP addition on the Raman spectrum of shrimp MPG. (A) effects of ADA and HPDSP addition on the Raman spectrum of shrimp MPG. (B) effects of ADA and HPDSP addition on the intensity of I_760_. (C) effects of ADA and HPDSP addition on the ratio of I_850_/I_830_. (D) effects of ADA and HPDSP addition on the relative content of protein *sec*ondary structure.

The intensity of Roman bands mainly reflected the changes in the secondary structure of MP, the effect of ADA and HPDSP additions on Roman spectra of MPG were shown in Fig. 4A. The assignment of the corresponding bands is included in Table S2 as described by Xu, Han, Fei, and Zhou (2011). The disulfide bond and sulfhydryl group play important roles in gel formation, and their absorption bands are in the range of 500–650 cm^−1^. Xu et al. (2011) reported that this Raman band almost disappeared at high temperature and shifted to lower frequency with the increased intensity. It could be observed that the band at 500–545 cm^−1^ became stronger, while the absorption peak at 642 cm^−1^ disappeared, suggesting that the S—S gauche conformation was dominant in the protein gel network. However, ADA or HPDSP did not affect the absorption strength of this range.

The stretching vibration of the tryptophan residue ring was located at a band near 760 cm^−1^. The intensity of I_760_ was related to hydrophobicity and aggregation of proteins, whereas the ratio of I_850_/I_830_ was associated with the exposure of tyrosine residues (Sánchez-González et al., 2008). The 1003 cm^−1^ bond was considered to be invariable in conformational changes of proteins, which was applied as a standard for the Raman band to do normalization.

The intensity of I_760_ decreased when the tryptophan residues of the protein were exposed to a polar microenvironment. As shown in Fig. 4B, the intensity of I_760_ decreased gradually with the increase of HPDSP addition. However, the intensity of I_760_ in 0.5 % and 1.0 % ADA groups were higher than that of the control group for the swelling of ADA changed the polar environment around the tryptophan residues.

The natural structure of proteins is highly folded. Heating could cause protein unfolding, promote the formation of various forces between the exposed groups, and form a three-dimensional network structure through the disulfide bond, non-disulfide covalent bond or hydrophobic interaction. It was reported that the beginning of protein denaturation may correspond to the cleavage of disulfide bonds. Similarly, the exposure of tyrosine residues (located at bonds 830 and 850 cm^−1^) also represents the unfolding of protein. If the ratio of I_850_/I_830_ is within the range of 0.7–1.0, the tyrosine residues are buried and hidden in the peptide chain. Conversely, the tyrosine residues are exposed on the protein surface and usually form weak or moderate hydrogen bonds when the ratio of I_850_/I_830_ is higher than or equal to 1.0 (Cortez-Trejo et al., 2022).

As shown in Fig. 4C, the ratio of I_850_/I_830_ was all higher than 1.0, representing that the proteins were unfolding and both ADA and HPDSP could promote the tyrosine residues to be exposed on the protein surface at high addition, while the addition of 0.5 % ADA, 1.0 % ADA and 0.5 % HPDSP had no significant effect on the ratio of I_850_/I_830_. The unfolded protein exposed hydrophobic groups and ordered the structure of proteins continued to unfold, increasing disordered structures (Mao et al., 2022). The results in Table 1 and Fig. 4C showed that as the hardness value increased, the ratio of I_760_ also increased; additionally, as the springiness value increased, the ratio of I_850_/I_830_ also increased. This indicated that hydrophobic interactions would affect the textural properties of MPG.

The amide I bond (1600–1700 cm^−1^) of proteins is useful in investigating protein secondary structure (Zhang et al., 2017), involving C=O stretching, C—N stretching, Cα-C-N bending and N—H in-plane bending of peptide groups. In general, the central bands of α-helix and β-sheet structures were in the range of 1645–1657 cm^−1^, and 1665–1680 cm^−1^, respectively, while the random coil was around 1660 cm^−1^. As shown in Fig. 4D, there was no significant effect on the relative content of the protein secondary structure with different additions of ADA or HPDSP, which indicated that the modified starch was filled in the network of the protein. Li et al. (2023) reported that the structure of α-helix could inhibit water retention, and the structure of β-folds (β-sheet and β-turn) contribute to the formation of favorable protein gels. The relative secondary structure content of β-folds in MPG is shown in Table S3, the 1.0 % HPDSP group had the highest content (71.92 ± 5.82 %). Through the observation of modified starch particles (Fig. 2), the 0.5 % ADA group had the largest particle size and the smallest pore size and the particle size of the 1.0 % HPDSP group was more uniform, suggesting that the stable structure of MPG was not only related to the structure of β-folds, but also other structures, such as random coil structures.

The central band of amide I was around 1660 cm^−1^, indicating that the random coil was dominated in protein conformation. The correlation between the frequency of amide I bond and the amount of protein conformation types exhibited that the proportion of β-turns and random coil fraction increased and α-helix decreased during thermal induction (Xu et al., 2011). The unfolding of protein structure was conducive to the exposure of hydrophobic groups, thereby affecting the functional properties of MPG. Previous research also showed the same results (Mi et al., 2022a).

In the range of 2800–3500 cm^−1^, peptides and proteins showed C—H stretching vibrations, and the bonds near 2900 cm^−1^ represented the exposure of a polar side chain in the aqueous environment of the protein (Wu et al., 2018). The decrease in the strength of this bond might be explained by the unfolding of the α-helix during heating and exposure to hydrophobic amino acids (Xu et al., 2011). However, the addition of ADA and HPDSP had little effect on the peak shape of MPG.

### 3.5 Docking of myosin with ADA and HPDSP

The MP's functional characteristics are primarily determined by its myosin component. Consequently, alterations in MP activity can be elucidated through a more detailed examination of the variations in myosin's structural and functional attributes. Molecular docking is a computational technique used to predict the preferred orientation of one molecule (known as the ligand) when it binds to another molecule (known as the receptor) to form a stable complex. This technique is widely used in the fields of bioinformatics, drug discovery, and structural biology to understand the interactions between molecules and to design new drugs. However, studies on molecular docking of ADA or HPDSP with *P. vannamei* myosin are scarce at present. To explore the binding mechanism between ADA, HPDSP and myosin, Autodock vina was used for molecular docking.

#### 3.5.1 Evaluation of myosin modeling results

**Fig. 5:**
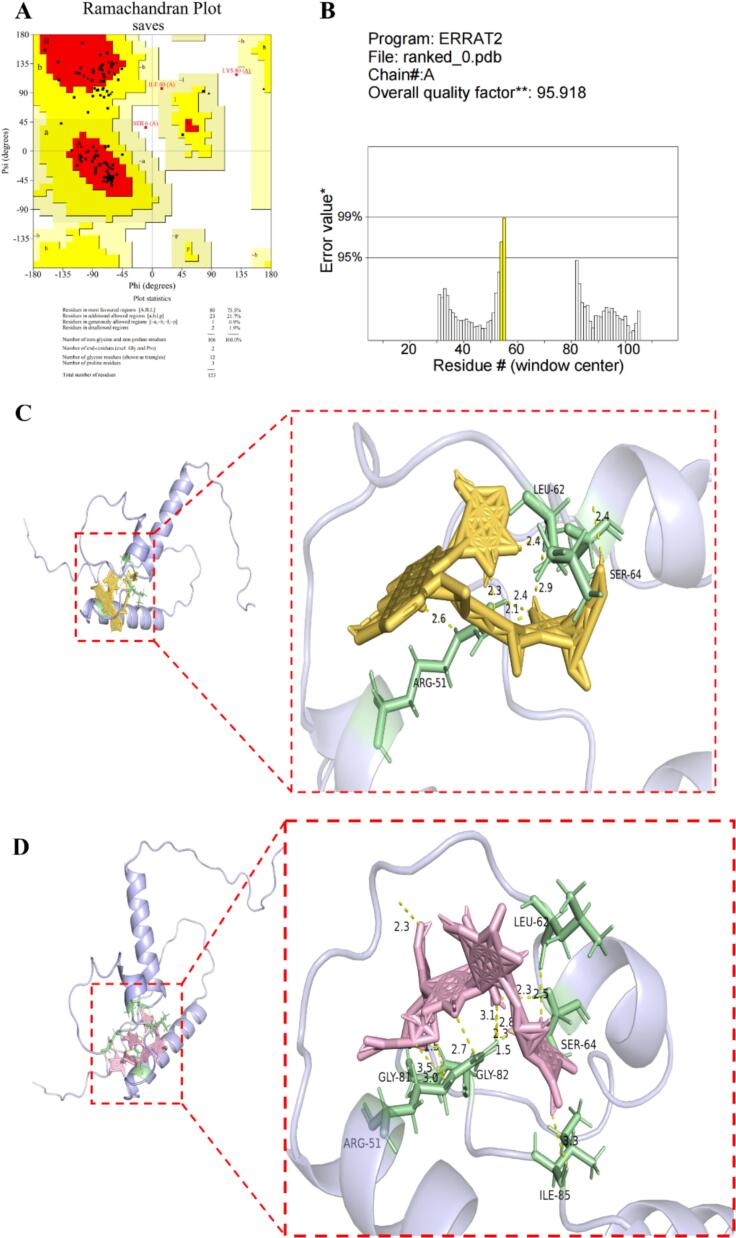
Molecular docking result of myosin with modified starches. (A) evaluation of myosin modeling with Ramachandran plot; (B) evaluation of myosin modeling with ERRAT; (C) molecular docking result of myosin with ADA; (D) molecular docking result of myosin with HPDSP.

Based on the Ramachandran plot analysis of the three-dimensional structure generated by Alphafold, it was observed that 75.5 % of the amino acid residues resided in the most favored regions. Furthermore, 21.7 % were situated in the additionally allowed regions, 0.9 % were positioned in the generously allowed regions, and 1.9 % were present in the disallowed regions, as depicted in Fig. 5A, and these residues will not affect the interaction of binding sites. The precision of the three-dimensional model was calculated to be 95.92 %, which attests to the model's adequacy for representing myosin, as illustrated in Fig. 5B. Consequently, this model is deemed appropriate for further exploration in molecular docking studies.

#### 3.5.2 Molecular docking

The molecular docking results indicate that both ADA (as shown in Fig. 5C) and HPDSP (as shown in Fig. 5D) are nested within the structural cavity of myosin, thus providing a molecular basis for the stability of the protein-modified amyloid system. The interactions between ADA, HPDSP and myosin are mainly mediated through hydrogen bonds involving key amino acids residues such as Leu 62, Ser 64, Arg 51 (Gly 81 and Ile 85 were only found in HPDSP). The results of Jiang et al. (2024) showed that the interaction between acetylated distarch phosphate -myosin (*Larimichthys crocea*) mainly involves hydrogen bonding (Leu 16, Cys 107, Glu 97 and Gly 101). In addition, acetylated distarch phosphate could fill the myosin gel network and maintain the gelation properties of myosin, which was beneficial to the functional and physicochemical properties of MP gels, consistent with the results of the present study. Thus, Leu 62, Ser 64, Arg 51 may be a major binding site for ADA, HPDSP and *P. vannamei* myosin, suggested that the starch-protein interaction was not merely a physical filling effect, but may involve specific, competitive binding at the myosin surface, potentially influencing myosin's aggregation behavior during gelation. However, molecular docking was simulated under unified docking parameters, and future studies using molecular dynamics simulations could provide a deeper understanding of the dynamic evolution of intermolecular forces in more complex, multi-component systems.

## 4 Conclusion

In this study, ADA and HPDSP significantly changed the dynamic rheological and structure characteristics of shrimp MPG. Although lower additions of ADA or HPDSP performed well in certain parameters, the 1.0 % ADA or 1.0 % HPDSP provided the most favorable overall performance. It delivered high G′ value, desirable springiness, and a homogeneous microstructure with uniform starch distribution, thereby ensuring a balanced enhancement in gel strength, structural integrity, and quality of shrimp surimi gels. The ADA and HPDSP exhibited different characteristics in MPG depending on their hydrophilic groups and the limited swelling ability of ADA or HPDSP could promote the MPG structure stronger. However, real food systems are more complex, and the influence of other components, such as salt (which enhances protein solubility), carbohydrates (which compete for water and affect gelation), and lipids (which enhances flavor and taste), remains to be investigated. Modified starches could form a stable composite gel, they could help maintain desired texture and juiciness, enabling a significant reduction in salt content without sacrificing product quality. Meanwhile, the reinforced structure by modified starch could mimic the smooth and creamy texture typically provided by fat, thereby improving the palatability of reduced-fat surimi gels. Therefore, the use of modified starches presents a viable pathway for formulating low-fat shrimp surimi products that still meet consumer expectations for texture.

## CRediT authorship contribution statement

**Jie Mi:** Writing – original draft, Software, Investigation, Formal analysis, Data curation. **Miao Jin:** Investigation. **Gaoshang Li:** Investigation. **Dalun Xu:** Writing – review & editing. **Ru Jia:** Writing – review & editing. **Huamao Wei:** Writing – review & editing. **Xiaojun Zhang:** Investigation. **Guangming Mei:** Investigation. **Wenge Yang:** Writing – review & editing, Project administration, Methodology, Funding acquisition.

## Declaration of competing interest

The authors declare that they have no known competing financial interests or personal relationships that could have appeared to influence the work reported in this paper.

## Data Availability

Data will be made available on request.
