# Supplementary material for: Mechanisms and physicochemical characteristics of modified starches in shrimp (Penaeus vannamei) myofibrillar protein gels
Source: Food Chem X. 2026 Jan 5;33:103496. doi: 10.1016/j.fochx.2026.103496 (PMC13216740; doi:10.1016/j.fochx.2026.103496)
Supplement: Supplementary file 1 [file mmc1.docx]

Table S1 the mean size of modified starch and pore size in MPG

| Addition (%) | | Mean size (μm) of  the modified starches | Mean size (μm) of  the pores |
| --- | --- | --- | --- |
| Control | 0.0 | / | 245.98±151.95^bcd^ |
| ADA | 0.5 | 184.35±63.78^a^ | 157.15±42.81^e^ |
|  | 1.0 | 139.72±41.84^bc^ | 182.30±88.49^de^ |
|  | 1.5 | 131.84±51.80^bc^ | 221.15±97.89^de^ |
|  | 2.0 | 137.87±59.61^bc^ | 231.24±72.86^cd^ |
|  | 2.5 | 161.20±85.79^b^ | 247.15±132.40^bcd^ |
| HPDSP | 0.5 | 127.36±48.37^c^ | 295.81±108.65^abc^ |
|  | 1.0 | 149.12±59.99^bc^ | 308.37±132.19^ab^ |
|  | 1.5 | 147.39±54.47^abc^ | 309.49±129.50^ab^ |
|  | 2.0 | 161.66±59.61^b^ | 362.97±196.10^a^ |
|  | 2.5 | 147.77±45.34^abc^ | 312.19±159.86^ab^ |

Values are presented as mean±SD. Different letters in the same column mean significant difference (*p* < 0.05).

The mean particle size of modified starches and ice crystals were observed at different magnification and they cannot be directly compared.

Table S2 Assignment of some Raman bonds.

| Wavenumber (cm^-1^) | Assignment |
| --- | --- |
| 514 | ν S-S gauche-gauche conformation |
| 530 | ν S-S gauche-gauche-trans conformation |
| 547 | ν S-S trans-gauche-trans conformation |
| 630-670 | ν S-S gauche conformation |
| 825, 852 | Tyr ν-ring |
| 758, 882 | Trp ν-ring |
| 932 | νCC α-helix |
| 1003 | Pheν-ring |
| 1034 | Pheν-ring |
| 1063 | ν-CN, ν-CH |
| 1126 | ν-CN |
| 1244 | Amide Ⅲ(β-helix) |
| 1302 | Amide Ⅲ(α-helix) |
| 1322, 1340 | δCH |
| 1410 | νCOO- Asp, Glu |
| 1450 | δasCH_3_, δCH_2_, δCH |
| 1600-1700 | Amide I |
| 2936 | νCH_3_, νCH_2_, νCH |

Table S3 The relative secondary structure content of β-folds in MPG

| Addition (%) | | β-folds (%) |
| --- | --- | --- |
| Control | 0.0 | 64.00±2.69^ab^ |
| ADA | 0.5 | 64.90±1.33^ab^ |
|  | 1.0 | 61.37±4.22^b^ |
|  | 1.5 | 65.59±2.29^ab^ |
|  | 2.0 | 64.79±0.87^ab^ |
|  | 2.5 | 64.72±2.21^ab^ |
| HPDSP | 0.5 | 63.13±4.59^b^ |
|  | 1.0 | 71.92±5.82^a^ |
|  | 1.5 | 67.79±6.39^ab^ |
|  | 2.0 | 66.90±2.65^ab^ |
|  | 2.5 | 62.90±2.36^b^ |

Values are presented as mean±SD. Different letters in the same column mean significant difference (*p* < 0.05).
